# Editorial: The Tumor Necrosis Factor Superfamily: An Increasing Role in Breast Cancer

**DOI:** 10.3389/fonc.2020.622588

**Published:** 2020-12-22

**Authors:** Dirk Geerts, John K. Cusick, Linda Connelly

**Affiliations:** ^1^ Department of Medical Biology, Amsterdam University Medical Center, Amsterdam, Netherlands; ^2^ Department of Basic Science, College of Medicine, California Northstate University, Elk Grove, CA, United States; ^3^ School of Medicine, California University of Science and Medicine, Colton, CA, United States

**Keywords:** tumor necrosis factor, breast cancer, SPP1/OPN, APRIL (TNFSF13), BAFF (TNFSF13B), osteoprotegerin (TNFRSF11B), receptor activator of nuclear factor-kB ligand (TNFSF11), TNF receptor superfamily

The Tumor Necrosis Factor (TNF) superfamily (TNFSF) comprises TNFSF ligands and their cognate TNF receptor superfamily (TNFRSF) members. TNFSFs are essential for innate and adaptive immunity and are implicated in diverse pathological conditions such as autoimmune diseases, developmental abnormalities, and cancer (1, 2). TNFSFs activate signaling pathways that mediate pro-tumor effects like inflammation and cell survival, and anti-tumor processes like apoptosis (3). Many TNFSFs have a role in breast cancer biology. The aim of this Research Topic is to increase understanding of TNFSF roles in breast cancer and provide insight into targeting this pathway for cancer treatment.

Liang et al. investigated single nucleotide variants (SNPs) in the Secreted phosphoprotein 1 (SPP1, or osteopontin) gene in breast cancer. Osteopontin, an extracellular matrix protein locally produced by multiple tissues but also secreted into the bloodstream, is a pro-inflammatory cytokine in bone mineralization and calcification. A negative regulator of TNFRSF5 (CD40), it was identified as a tumor-promoting protein. It is over-expressed in several different cancers, and is linked to tumor progression in ovarian cancer, poor prognosis in colorectal cancer, and cell migration, stemness, and cancer risk in breast cancer. SPP1 SNPs have been associated with cancer risk or metastasis in breast, liver, and oral cancer. Liang et al. now find that the CC genotype for SPP1 SNP rs11730582, located in its promoter, is correlated to decreased risk for breast cancer, and is more prevalent in breast cancer patients with stage I–III, than with stage IV disease, when compared with the TT phenotype. Interestingly, they also found that plasma SPP1 levels are lower in patients and healthy control subjects with the CC genotype, in support of a tumor-promoting role for SPP1 in breast cancer.

Originally named for its ability to kill tumors, the pro-inflammatory cytokine TNFα acts in initiation and progression of many cancers, including breast cancer. Mercogliano et al. describe the complex activity of TNFα, which functions as a secreted (sTNFα) or transmembrane protein, on TNFR1 and TNFR2. Although TNFα induces apoptosis in breast cancer cell lines, many cancers *in vivo* become resistant to TNFα-mediated cytotoxicity. Furthermore, TNFα can contribute to progression of multiple breast cancer subtypes by stimulating the pro-survival transcription factors c-Jun and NF-κB. Additional pro-metastatic properties of sTNFα in breast cancer include matrix metalloproteinase upregulation and HER2 receptor transactivation. The authors discuss an immunomodulatory role for TNFα in protecting cancer from the immune system. Lastly, they describe the potential benefit of combining sTNFα targeting with Trastuzumab, as sTNFα antagonists block mucin MUC4 expression, making HER2 more accessible to Trastuzumab. Similarly, the authors discuss the potential benefit of combining TNFα antagonists and immune checkpoint inhibitors to prevent resistance to immune checkpoint blockade.

Kampa et al. focus on the family of TNFRSF members associated with B-cell development and function, BCMA (TNFRSF17), BAFFR (TNFRSF13C), and TACI (TNFRSF13B) and their ligands APRIL (TNFSF13) and BAFF (TNFSF13B). APRIL and BAFF both bind BCMA and TACI with differing affinities but BAFF only binds BAFFR. The authors discuss these signaling networks in B cell maturation, survival, and differentiation into plasma cells. In addition, the authors describe high BCMA and TACI receptor expression in other hematopoietic lineages, such as T lymphocytes and macrophages, indicating that APRIL and BAFF may act in the immune system’s contribution to the tumor microenvironment in cancers such as breast cancer. All three TNFRs can prevent apoptosis by upregulating the pro-survival transcription factor NF-κB and decreasing levels of pro-apoptotic Bim. The authors also discuss BAFF and APRIL signaling in hematologic malignancies and the potential for targeting these pathways for the treatment of solid tumors such as breast cancer. They describe the expression of these ligands and receptors in breast cancer and propose further examination of APRIL and its receptors in breast cancer as APRIL can promote the survival, proliferation and migration of breast cancer cells. Finally, the authors discuss the potential for targeting BCMA, since its over-expression in breast cancer correlates with that of the checkpoint molecule PDL-1, yet caution against potential drawbacks of anti-BCMA therapy.

Receptor activator of nuclear factor kappa-B ligand (RANKL; TNFSF11), its receptor RANK (TNFRSF11A) and its decoy receptor Osteoprotegerin (OPG; TNFRSF11B) stand out as key TNFSFs in breast cancer. Cronin et al. describe their signaling in bone remodeling and the immune system as an introduction to subsequent studies on breast cancer. They describe how RANKL is involved in murine mammary gland development, the role of RANKL in breast tumor progression, and recent studies linking RANKL/RANK signaling to BRCA1-mutation driven breast cancer. The authors discuss the potential for targeting this pathway by the monoclonal antibody Denosumab which blocks RANK/RANKL interactions as chemoprevention in BRCA1 mutation carriers, and to inhibit bone metastases in advanced disease. They describe current clinical data in breast and other cancers, highlighting that this is a very active area of interest in breast cancer.

While Ming et al. focus on RANKL/RANK the review by Geerts et al. looks at the less-characterized member of this signaling trio. A growing body of studies reports on the relationship between OPG expression, breast cancer risk and prognosis. They note that OPG, which binds and inhibits RANKL, could also block another TNFSF; TNF-Related Apoptosis Inducing Ligand (TRAIL; TNFSF10). While OPG-RANKL binding could be anti-tumorigenic, OPG-TRAIL could be tumor-promoting. Indeed, Geerts et al. describe that OPG expression is linked with increased and lowered risk of breast cancer development, and with poor or good prognosis in breast cancer patients. The authors identify varying analyses of OPG mRNA versus serum protein levels and differences due to subtype (Estrogen positive or negative). They conclude that the clinical data align with reports of both pro- and anti-tumor effects of OPG from basic research studies and may reflect the different interactions of OPG with RANKL or TRAIL.

Overall, these studies highlight the growing link between the TNF superfamily and breast cancer and the potential for specific targeting of TNFSF’s in novel clinical applications. This could be through biomarker development or targeting of specific family members such as sTNFα and RANK/RANKL alone or in combination with existing therapies.

## Author Contributions

DG, JC and LC jointly conceptualized, wrote and edited the manuscript. All authors listed have made a substantial, direct, and intellectual contribution to the work and approved it for publication.

## Conflict of Interest

The authors declare that the research was conducted in the absence of any commercial or financial relationships that could be construed as a potential conflict of interest.
